# A Novel System for Transcutaneous Application of Carbon Dioxide Causing an “Artificial Bohr Effect” in the Human Body

**DOI:** 10.1371/journal.pone.0024137

**Published:** 2011-09-08

**Authors:** Yoshitada Sakai, Masahiko Miwa, Keisuke Oe, Takeshi Ueha, Akihiro Koh, Takahiro Niikura, Takashi Iwakura, Sang Yang Lee, Masaya Tanaka, Masahiro Kurosaka

**Affiliations:** 1 Department of Orthopedic Surgery, Kobe University Graduate School of Medicine, Kobe University, Kobe, Hyogo, Japan; 2 NeoChemir Inc., Kobe, Hyogo, Japan; 3 Faculty of Health Care Sciences, Himeji Dokkyo University, Himeji, Hyogo, Japan; University of Pittsburgh, United States of America

## Abstract

**Background:**

Carbon dioxide (CO_2_) therapy refers to the transcutaneous administration of CO_2_ for therapeutic purposes. This effect has been explained by an increase in the pressure of O_2_ in tissues known as the Bohr effect. However, there have been no reports investigating the oxygen dissociation of haemoglobin (Hb) during transcutaneous application of CO_2_ *in vivo*. In this study, we investigate whether the Bohr effect is caused by transcutaneous application of CO2 in human living body.

**Methods:**

We used a novel system for transcutaneous application of CO_2_ using pure CO_2_ gas, hydrogel, and a plastic adaptor. The validity of the CO_2_ hydrogel was confirmed *in vitro* using a measuring device for transcutaneous CO_2_ absorption using rat skin. Next, we measured the pH change in the human triceps surae muscle during transcutaneous application of CO_2_ using phosphorus-31 magnetic resonance spectroscopy (^31^P-MRS) *in vivo.* In addition, oxy- and deoxy-Hb concentrations were measured with near-infrared spectroscopy in the human arm with occulted blood flow to investigate O2 dissociation from Hb caused by transcutaneous application of CO_2_.

**Results:**

The rat skin experiment showed that CO_2_ hydrogel enhanced CO_2_ gas permeation through the rat skin. The intracellular pH of the triceps surae muscle decreased significantly 10 min. after transcutaneous application of CO_2_. The NIRS data show the oxy-Hb concentration decreased significantly 4 min. after CO_2_ application, and deoxy-Hb concentration increased significantly 2 min. after CO_2_ application in the CO_2_-applied group compared to the control group. Oxy-Hb concentration significantly decreased while deoxy-Hb concentration significantly increased after transcutaneous CO_2_ application.

**Conclusions:**

Our novel transcutaneous CO_2_ application facilitated an O_2_ dissociation from Hb in the human body, thus providing evidence of the Bohr effect *in vivo*.

## Introduction

Carbon dioxide (CO_2_) therapy refers to the transcutaneous or subcutaneous administration of CO_2_ for therapeutic purposes especially in the treatment of peripheral vascular disorder [1]. One example of this is the use of spa therapy that emerged as an important treatment in Europe during the 1800 s and is still in use in many countries today [2]. Another example is the use of artificial CO_2_ enriched water for bathing, which has been clinically applied to improve ischemic limb symptom [3]–[5]. In plastic surgery, subcutaneous injection of CO_2_ is applied if skin irregularity and/or adiposity occurs [6], [7]. Recently, some reports showed that the transcutaneous administration of CO_2_ rich spa gas improves microcirculation and symptoms in patients who have intermittent claudication [8], [9] and Raynoud's phenomenon [10]. These therapeutic effects of CO_2_ are caused by an increase in blood flow and microcirculation assessed by Laser Doppler [10], and an increase of tcPO_2_ in ischemic tissues, which is explained by the Bohr effect [3], [5], [8], [9], [11].

The Bohr effect is represented by a rightward shift of the O_2_–Hb dissociation curve with an increase in pCO_2_ or decrease in pH [12]–[15]. It has frequently been studied in physiology. However, subjects' blood samples were used in these previous experiments, and the O_2_ affinity of Hb measured only in vitro or ex vivo. In addition, although the Bohr effect has often been referred to as an explanation for the therapeutic usefulness of CO_2_ therapies, no reports have actually provided evidence for the Bohr effect in CO_2_ therapies as well as evidence for the transcutaneous absorption of CO_2_. In addition, to the best of our knowledge, there have been no reports that have investigated the Bohr effect in vivo.

To produce the effect of CO_2_ therapy, an adequate amount of CO_2_ needs to be delivered to local tissues without difficulty and invasion. Previously, only three methods of CO_2_ therapy have been reported that have been able to accomplish this. The first method requires bathing in CO_2_-enriched water such as in a carbonated spa [2], or artificially carbonated water prepared by the chemical reaction of succinic acid and sodium bicarbonate [3], [4], or by blowing micro-bubbles into the water through a CO_2_ gas-permeable membrane [5], [16]–[19]. However, the CO_2_ concentration of saturated CO_2_-enriched water is only 0.1% [16]–[18] and there is no evidence of CO_2_ absorption into the human body. The second method is direct subcutaneous CO_2_ injection [6], [7]. Even though direct subcutaneous CO_2_ injection can deliver pure CO_2_ into local tissues, this method is invasive, involves an infection risk, and is difficult to use over a large area of the body.

The third method is the transctaneous administration of CO_2_ natural spa gas [8]–[10]. Previous reports have outlined this method of transctaneous administration of CO_2_ natural spa gas into the whole limb as follows: The subjects take a bath, humidify their skin and then CO_2_ gas is administered transcutaneously by covering the subject's body with a large bag. This method administrates an adequate CO_2_ concentration, however, it is difficult to obtain CO_2_ natural spa gas, and a large space is needed to set up the bath.

**Figure 1:**
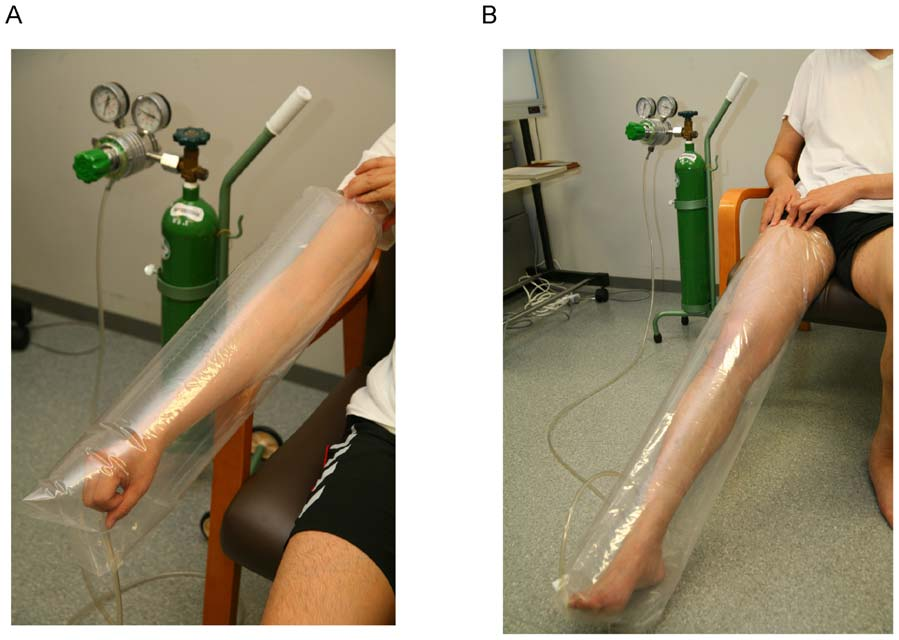
Rationale of the experimental outline. (A) The application of our novel system for transcutaneous application of CO_2_ (For the upper limb). (B) The application of our novel system for transcutaneous application of CO_2_ (For the lower limb).

To solve these problems, we designed a novel transcutaneous CO_2_ application system using 100% CO_2_ gas, a transcutaneous CO_2_ absorption-enhancing hydrogel (CO_2_ hydrogel) and a CO_2_ adaptor that seals the body surface and traps the gas inside (Fig. 1, A and B). In this system, the CO_2_ hydrogel is applied to the skin to allow CO_2_ to dissolve and penetrate into the local tissue, which humidifies the skin without bathing, thereby forming a passage for CO_2_ to reach the local tissues. This system allows for the easy application of CO_2_ to any site of the body. In addition, the system provides for simple sterilization and is not invasive.

In this study, we investigated whether our transcutaneous CO_2_ application system caused CO_2_ absorption into local tissues and the Bohr effect in the human body, by real time and non-invasive measurement of changes in pH and oxygenated and deoxygenated-hemoglobin volume.

## Methods

### Novel system for transcutaneous CO_2_ application

A set of measuring devices for transcutaneous CO_2_ absorption, a plastic CO_2_ adaptor and transcutaneous CO_2_ absorption-accelerating hydrogel, (Formulation: carbomer (0.65%), glycerin (5.00%), sodium hydroxide (0.18%), sodium alginate (0.15%), sodium dihydrogen phosphate (0.15%), methylparaben (0.10%), and deionized water (balance)) were obtained from NeoChemir Inc., Kobe, Japan (International patent publication number: WO2004/002393). Pure CO_2_ gas was purchased from Kobe Sanso Inc., Kobe, Japan. The actual application to humans is shown in Figures 1A and 1B. When we applied CO_2_ transcutaneously using this system, sweating and redness of the skin were noted. In addition, the blood flow to the fingers increased, as shown by a Laser Doppler study (Data not shown).

### Validation of transcutaneous CO_2_ absorption accelerating hydrogel using a measuring device for transcutaneous CO_2_ absorption

Subjects: Six Sprague–Dawley rats were purchased (CLEA Japan, Tokyo, Japan). The animal experiment plan was reviewed and approved by the Animal Research Committee of Kobe University Graduate School of Medicine. The approval ID is P00220.

**Figure 2:**
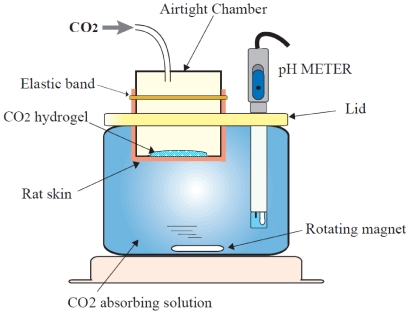
Measuring device to validate CO_2_ hydrogel *in vitro* using rat skin.

A set of measuring devices for transcutaneous CO_2_ absorption consisting of (1) a container filled with CO_2_ absorbing solution (600 mL of pure water) with a pH meter (D52: HORIBA, Kyoto, Japan) and a magnetic stirrer and (2) an airtight CO_2_ gas chamber, with a 5-cm diameter hole covered by a skin specimen positioned at the bottom of the chamber, was used to observe the permeability of CO_2_ gas thorough the rat skin specimen covered with, or without, the CO_2_ hydrogel. (Figure 2) The CO_2_-absorbing solution absorbs CO_2_ gas through the skin specimen, and the pH of the solution decreases depending on the volume of the absorbed CO_2_ (H_2_O + CO_2_ → H^+^ + HCO_3_^−^). The skin from depilated Sprague–Dawley rats was harvested in 10×10-cm sections. Immediately after harvesting, a skin specimen was positioned over the hole of the chamber, in contact with the CO_2_-absorbing solution, the depilated surface facing upwards. CO_2_ hydrogel (0.5 g) was applied to the skin specimen in the Gel (+) groups but not in the Gel (−) groups, and the chamber was filled with pure CO_2_ gas. After filling, the pH of the solution was measured in 30-s intervals for 15 min. by a pH meter.

### Measurement of intramuscular pH in vivo using phosphorus-31 magnetic resonance spectroscopy (^31^P-MRS) during transcutaneous application of CO_2_ in vivo

Subject: Five healthy male volunteers with no history of respiratory or vascular disease participated in this study. Subjects were 23–38 years old (average: 33.0±6.6). This study was approved and permitted by the Ethical Committee of Kobe University Graduate School of Medicine and informed consent was obtained by written from all subjects before the start of the study. The approval ID is 997.

^31^P-MRS: The intramuscular pH was measured in the triceps surae muscle. All MR studies were performed with a 1.5-T superconducting imaging system (Gyroscan NT-Intera; Philips Medical Systems, Best, The Netherlands) and a surface coil. CO_2_ hydrogel was applied to the subject's lower leg. A plastic CO_2_ adaptor was then attached to the subject's lower leg, and the surface coil was positioned over the adaptor. After measurement preparations, pure CO_2_ gas was flowed into the adaptor. Data were collected before infusion of CO_2_ and every 5 min. after infusion. Quantification of the ^31^P-MRS metabolite data was reported before [20], and pHi was determined from the chemical shift of Pi with respect to PCr.

### Measurement of oxygenated and deoxygenated Hb concentration during transcutaneous application of CO_2_ in vivo

Subjects: Seven healthy male volunteers with no history of respiratory or vascular disease participated in this study. Subjects were 27–40 years of age (average: 32.0±4.6). This study was approved and permitted by the Ethical Committee of Kobe University Graduate School of Medicine and informed consent was obtained by written from all subjects before the start of the study. The approval ID is 619.

A near-infrared spectroscopy (NIRS), (NIRO-200 with multi-fiber adaptor: Hamamatsu Photonics. K. K. Hamamatsu, Japan), was used for Hb concentration measurement. Changes in oxygenated and deoxygenated Hb concentrations were measured using 3 channels by focusing on the differences in absorption of light at 775, 810, and 850 nm [21]–[23]. The recording probe was attached to the inner side of the subject's forearm. A pneumatic tourniquet system (ATS2000, Zimmer patient care division, Dover, OH) commonly used in orthopaedic surgery was used for avascularization of the arm.

**Figure 3:**
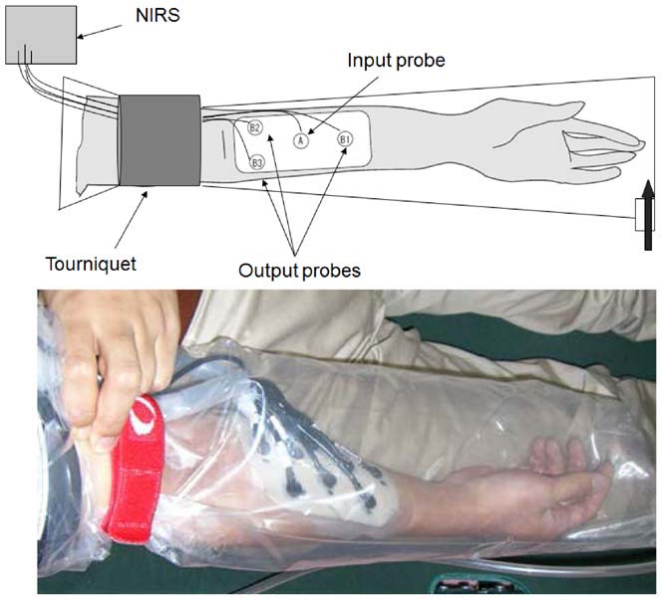
Novel system for transcutaneous CO_2_ application in the forearm with NIRS probe.

Each subject entered the environmental chamber, which was maintained at an ambient temperature of 26°C and relative humidity of 45%. NIRS probes were attached to the subjects' forearms. A tourniquet was wound around the upper arms, and then Hb concentration was measured. After confirming that the oxy-/deoxy-Hb ratio had stabilized, the tourniquet was inflated to a pressure of 250 mmHg, a commonly used pressure level in surgeries to avoid bleeding from the forearm (Figure 3). Eight minutes after the inflation, CO_2_ hydrogel was applied to the subject's forearm, and the entire arm was enclosed by a CO_2_ adaptor. Ten minutes after the inflation, pure CO_2_ gas or air (control) was allowed to flow into the adaptor. The relative concentrations of the oxy- and deoxy-Hb were measured at 2-s intervals using NIRS. The duration of tourniquet inflation was limited to a maximum of 20 min to avoid ischemic damage to subjects' tissues.

### Statistical analysis

Paired *t*-tests were used to compare all the variables in the control and CO_2_ groups. All values were analysed with measurement analysis of variance (ANOVA), followed by analysis of simple main effects. All data are presented as mean ± S.E.M. *P*<0.05 was considered statistically significant.

## Results

### Validation of transcutaneous CO_2_ absorption accelerating hydrogel using a measuring device for transcutaneous CO_2_ absorption

**Figure 4:**
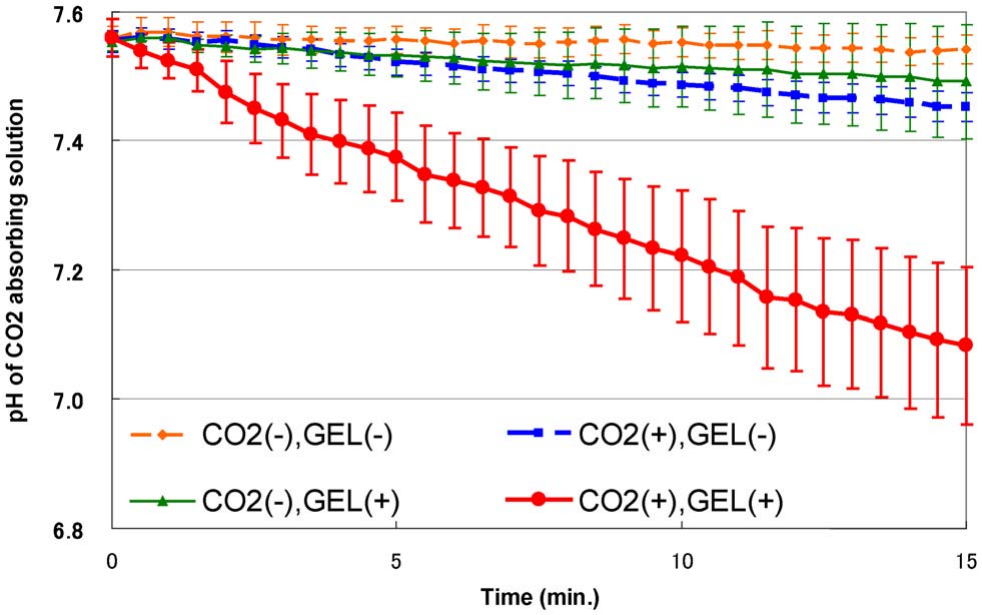
pH changes in CO_2_-absorbing solution during transcutaneous CO_2_ application through the rat skin with or without the CO_2_ hydrogel. The graph shows pH changes during CO_2_ application with or without the CO_2_ hydrogel. (n = 6). Graph data are expressed as means ± S.E.M.

Before we used this system in humans, the validity of the CO_2_ hydrogel was confirmed *in vitro* using a measuring device for transcutaneous CO_2_ absorption (Figure 2). Four groups of rat skin specimens with or without CO_2_ hydrogel that had been filled with CO_2_ gas or air were used in the experiment. The CO_2_-absorbing solution receives CO_2_ gas through the skin specimen, and the pH of the solution decreases, depending on the volume of the absorbed CO_2_. The pH of the solution decreased time-dependently in the CO_2_ (+) groups, and the pH values were significantly lower in the CO_2_ (+) Gel (+) group compared to the CO_2_ (+) Gel (−) group after 3.5 min (Figure 4). These results showed that CO_2_ hydrogel actually enhanced CO_2_ gas permeation through the rat skin.

### Measurement of intramuscular pH in vivo using ^31^P-MRS during transcutaneous application of CO_2_ in vivo

**Figure 5:**
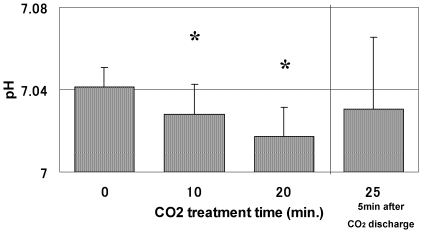
Intramuscular pH changes in the triceps surae muscle during transcutaneous application of CO_2_ using ^31^P-MRS. The graph shows that pH decreases during the accumulation of CO_2_. (n = 5) Graph data are expressed as means ± S.E.M.

Next, to test this system in the human body, we measured the pH change in the muscle during transcutaneous application of CO_2_. We used phosphorus-31 magnetic resonance spectroscopy (^31^P-MRS) to measure the intracellular acid–base status in the subject's triceps surae muscle [20]. The room temperature was 25°C. CO_2_ hydrogel was first applied to the subject's lower leg, and the CO_2_ adaptor was attached to seal the lower leg; then the surface coil was applied over the adaptor adhering it to the calf. After preparing the subject for measurement, pure CO_2_ was infused into the adaptor. The measurements were performed before CO_2_ infusion and every 5 min. after infusion. The intracellular pH of the triceps surae muscle decreased significantly 10 min. after transcutaneous application of CO_2_ (Figure 5). The results showed that the intramuscular pH decreased by transcutaneous application of CO_2_ using this system *in vivo*. Although the pH change was expected to be buffered by body fluid and active blood flow, buffering was not enough to prevent the pH change. From these results, we confirmed that our novel system allowed transcutaneous penetration of CO_2_ *in vivo*.

### Measurement of oxygenated and deoxygenated Hb concentration during transcutaneous application of CO_2_ in vivo

**Figure 6:**
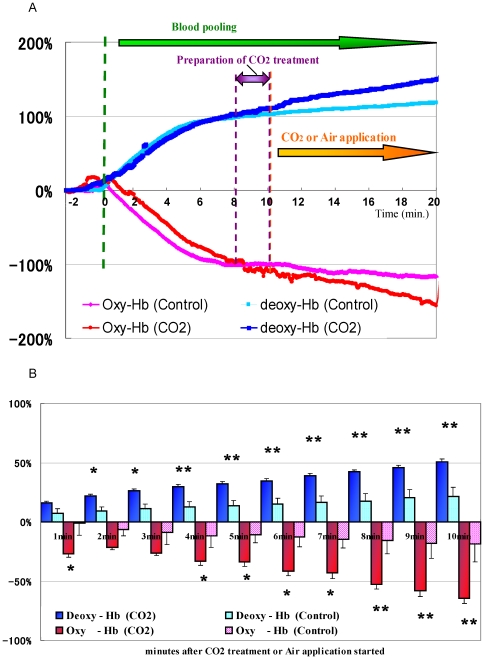
Measurement of oxygenated and deoxygenated Hb concentration during transcutaneous application of CO_2_ using NIRS. (A) Continuous measurement of oxy and deoxy-Hb concentrations using NIRS with pooling blood by a pneumatic tourniquet. The bold lines demonstrate the CO_2_ group data. All data show the changes in Hb concentrations from the starting point to the end point of measurement (n = 7). Data show the decrease in oxy-Hb and the increase in deoxy-Hb after pooling blood, followed by the greater decrease in oxy-Hb and greater increase in deoxy-Hb in CO_2_ group after transcutaneous CO_2_ application. (B) Relative changes in amounts of oxy/deoxy-Hb (the values 8 min. after blood pooling started were set as standards). Graph data are expressed as mean ± S.E.M. The averages and significance checks were calculated based on measurements of the 7 subjects. Statistical significance at P<0.05 is denoted by *, and P<0.01 is denoted by **. The graph shows a significant decrease in oxy-Hb and increase in deoxy-Hb in the CO_2_ group.

The oxy- and deoxy-Hb concentrations at all time points during the experiment are shown in Figure 6A. The oxy-Hb concentration decreased and the deoxy-Hb concentration increased after halting the blood flow (resting O_2_ consumption). The relative concentrations of oxy- and deoxy-Hb changed gradually, almost reaching a maximum after 8 min. Both the decrease in oxy-Hb and the increase in deoxy-Hb were greater in the CO_2_-applied arms than in the control arms.

Oxy- and deoxy-Hb concentrations at 8 min. after inflation were assigned to be the control values. The mean values of 5 points around every second minute of measurement are shown in Figure 6B and Table. 1. The data show that the oxy-Hb concentration decreased significantly 4 min. after CO_2_ application (The relative changes of the oxy-Hb concentrations were −33.4±23.9% in the CO_2_ applied group and −11.8±8.0% in the air applied (control) group), and that deoxy-Hb concentration increased significantly 2 min. after CO_2_ application in the CO_2_-applied group compared to the control group (The relative changes of the deoxy-Hb concentrations were 21.9±3.6% in the CO2 applied group and 9.1±1.6% in the control group). Thus, it was confirmed that transcutaneous CO_2_ application facilitates a decrease in oxy-Hb and an increase in deoxy-Hb in the human body, providing evidence of O_2_ dissociation of Hb resulting from transcutaneous application of CO_2_ *in vivo*.

**Table 1:** Calculated data showing relative changes in relative amounts of oxy/deoxy-Hb (values 8 min. after blood pooling started were set as standards).

Time		1 min	2 min	3 min	4 min	5 min	6 min	7 min	8 min	9 min	10 min
**Oxy - Hb**	**Control±S.E.M.**	-0.9%	-6.3%	-8.8%	-11.8%	-10.7%	-12.5%	-14.8%	-15.8%	-18.3%	-18.5%
		±2.7%	±2.2%	±2.0%	±3.3%	±4.1%	±3.9%	±4.9%	±4.2%	±5.0%	±4.7%
	**CO2±S.E.M.**	-26.9%	-21.3%	-26.4%	-33.4%	-33.6%	-41.2%	-43.0%	-52.4%	-58.0%	-64.2%
		±10.4%	±5.2%	±10.3%	±9.7%	±6.8%	±8.6%	±7.2%	±10.9%	±12.5%	±15.0%
	***P*** ** Value**	0.0169	0.1678	0.104	0.0466	0.0351	0.0086	0.0099	0.0009	0.0003	0
**Deoxy - Hb**	**Control±S.E.M.**	7.3%	9.1%	11.1%	12.7%	13.6%	15.2%	16.6%	17.7%	20.3%	21.4%
		±1.5%	±1.4%	±1.5%	±1.5%	±1.7%	±1.7%	±1.8%	±1.5%	±2.0%	±2.3%
	**CO2±S.E.M.**	16.3%	21.9%	26.3%	30.0%	32.4%	34.7%	39.1%	42.2%	46.0%	50.6%
		±3.8%	±3.6%	±4.1%	±4.3%	±4.6%	±4.8%	±5.4%	±6.1%	±6.8%	±7.7%
	***P*** ** Value**	0.1088	0.0223	0.0072	0.0023	0.0009	0.0006	0.0001	0	0	0

The data are expressed as mean ± S.E.M. The averages and significance levels were calculated based on the measurements of the 7 subjects. Statistical significance at *P*<0.05 is denoted by * and *P*<0.01 is denoted by **.

## Discussion

In this study, we showed that our transcutaneous CO_2_ system could cause the absorption of CO_2_, and the Bohr effect in the human body. A number of studies into the physiological effects of CO_2_ therapy, especially CO_2_-enriched water bathing [3]–[5], [16]–[19] and CO_2_ natural spa gas therapy [8]–[10] have been published. The effect of CO_2_ therapy for peripheral vascular disorder has been explained by the vasodilation effect by CO_2_^1,11^, and the Bohr effect [3], [5], [8]–[11]. For example, Hartmann et al demonstrated an increase in tissue oxygen pressure which was caused by CO_2_-enriched water bathing, and they concluded this increase was caused by the Bohr effect [3]. However, they showed no direct evidence for the Bohr effect in their study. To the best of our knowledge, there have been no reports that have investigated the Bohr effect in vivo.

The issue for successful investigation of the Bohr effect *in vivo* is that the O_2_ dissociation from Hb needs to be measured in an *in vivo* and real-time manner without taking blood samples. A study by Jöbsis demonstrated the feasibility of using near-infrared spectroscopy (NIRS) to assess the adequacy of O_2_ provision and utilization in living tissues [21]. NIRS can track changes in tissue oxy- and deoxy-Hb concentrations in a non-destructive, continuous, and real-time manner; thus, NIRS can be used to assess dynamic changes of the tissue oxy- and deoxy-Hb concentrations [22], [23]. In the present study, therefore, NIRS was used to confirm whether transcutaneous CO_2_ application actually causes O_2_ dissociation from the oxy-Hb, which is a characteristic phenomenon of the Bohr effect.

Another issue to be solved is that the blood flow flushes away CO_2_-absorbed erythrocytes *in vivo*. In our preliminary experiments, no changes were observed in oxy- and deoxy-Hb concentrations during transcutaneous CO_2_ application to the arm (data not shown). We hypothesized that this resulted from an active blood flow that caused an outflow of the deoxy-Hb enriched erythrocytes, which were then deoxygenated by the transcutaneously absorbed CO_2_, as well as by an inflow of the oxy-Hb enriched normal erythrocytes. Therefore, we employed a pneumatic tourniquet (commonly used in a number of surgeries) [24], [25] to halt the blood flow and keep the erythrocytes in the arm during the transcutaneous CO_2_ application. In addition, the pneumatic tourniquet reduced the problems inherent in measuring the O_2_ dissociation from the Hb, for example, by an increase in local tissue temperature caused by a hypercapnia-induced increase in blood flow. Thus, we believe this study can be regarded as the first to provide real evidence of the Bohr effect in the human body.

One potential flaw in this study is that NIRS measures not only Hb but also myoglobin [26], [27]. The O_2_ dissociation curve of myoglobin is a rectangular hyperbola, and myoglobin releases oxygen at a very low pO_2_ [27], as P_50_ of myoglobin is 2.03 mmHg at 35°C [28]. In a previous report, tissue pO_2_ was shown to be about 25–45 mmHg with blood pooling by applying a pneumatic tourniquet for 10–20 min [24], [25]. In addition, the relationship of myoglobin P_50_ with pH is linear [28]. In contrast, the relationship of Hb P_50_ with pH is exponential—the well-known Bohr coefficient [15], [29], [30]. Therefore, the contamination of myoglobin in NIRS measurement is expected to have only a minimal influence on the data.

CO_2_ therapy has a clinical effect in the treatment of ischemic legs and Raynoud's phenomenon. The effect is caused by the improvement of microcirculation, increasing in tcPO_2_, and causing the Bohr effect, which we report here. Our experimental results also show scientific evidence that our transcutaneous CO_2_ application can cause an “Artificial Bohr effect.” This artificial Bohr effect might be a potential new therapy for disorders in which a high quantity of O_2_ in local tissues is required for treatment as well as in the peripheral vascular disorder.
